# Emerging Environmental Contaminants Targeting Cardiovascular Ion Channels: Exposure Effects, Underlying Mechanisms, and Implications for Cardiovascular Health Risks

**DOI:** 10.3390/toxics14050450

**Published:** 2026-05-21

**Authors:** Dingshan Zhan, Dan Li, Shulin Guo, Xuyang Chai, Rongkai Cao, Weicong Deng, Kaihan Wu, Yu Li, Suk Ying Tsang, Zongwei Cai, Zenghua Qi

**Affiliations:** 1 Guangdong-Hong Kong-Macao Joint Laboratory for Contaminants Exposure and Health, School of Environmental Science and Engineering, Guangdong University of Technology, Guangzhou 510006, China; 2112207032@mail2.gdut.edu.cn (D.Z.); danlicmu@163.com (D.L.); guoshulin2024@163.com (S.G.); mayday1229236705@foxmail.com (X.C.); 3123005531@mail2.gdut.edu.cn (R.C.); 15089298326@163.com (K.W.); a13697470563@icloud.com (Y.L.); 2 School of Life Sciences, The Chinese University of Hong Kong, Hong Kong, China; fayetsang@cuhk.edu.hk; 3 Eastern Institute of Technology, Ningbo 315100, China

**Keywords:** emerging contaminants, cardiac ion channels, cardiovascular toxicity, arrhythmia, environmental exposure

## Abstract

Emerging contaminants (ECs) encompass a wide spectrum of pollutants, from endocrine disruptors and persistent organic pollutants to microplastics and pharmaceutical residues. These contaminants often exhibit distinct chemical and physical properties compared with traditional pollutants and potentially pose risks to human health, especially as they have become pervasive in environmental and biological systems. ECs can also pose a significant threat to cardiovascular health, as they may target the ion channels that are critical to regulating cardiac excitability and contraction. However, the impact of ECs on the cardiovascular system, particularly on cardiac ion channels, remains elusive. In this review, we aim to provide an overview of the knowledge base concerning the impact of emerging contaminants on cardiac ion channels, with an emphasis on the effects of these compounds on cardiac excitability, contractility, and overall cardiovascular function. We first outline the structural and functional characteristics of ion channels, along with how these transmembrane proteins regulate cardiac physiology. Subsequently, we detail how typical ECs directly or indirectly interact with various ion channels—including sodium, calcium, potassium channels, as well as ion transporters and exchangers. Special attention is given to studies that have demonstrated cell-level responses or examined how pollutant concentration and chemical structure affect the modulation of ion channels. This review compiles recent research reports to elucidate the mechanisms by which EC exposure disrupts cardiac ion channels, potentially leading to cardiotoxicity. Moreover, the insights gathered herein illuminate critical research gaps and outline essential directions for future investigations.

## 1. Introduction

A rapid expansion in the use of synthetic organic chemicals over recent decades, a direct result of industrialization and urbanization, has introduced a vast array of ECs into environmental and biological systems [1]. Emerging contaminants, also referred to as contaminants of emerging concern (CECs), are natural or synthetic chemicals. Although these compounds are not routinely monitored or fully regulated, they are increasingly detected in the environment and may thus pose potential risks to ecosystems and human health. The Chemical Abstracts Service registry, which had included 20 million chemicals in 2002, surged to 219 million chemicals by 2024; this growth reflects an unprecedented level of chemical diversity in contemporary society [2]. ECs are commonly categorized into four broad groups of global concern: persistent organic pollutants, endocrine-disrupting chemicals, antibiotics, and microplastics. However, these broad classifications encompass numerous chemically distinct contaminant classes with diverse physicochemical properties, environmental behaviors, and toxicological mechanisms. In particular, representative EC groups such as bisphenol A (BPA), per- and polyfluoroalkyl substances (PFAS), phthalates (PAEs), flame retardants (FRs), polycyclic aromatic hydrocarbons (PAHs), polychlorinated biphenyls (PCBs), pesticides, microplastics (MPs) and pharmaceuticals and personal care products (PPCPs) have recently attracted increasing attention because of their widespread environmental occurrence, high human exposure potential, and emerging cardiovascular toxicity [3,4]. It is important to note that cardiovascular diseases (CVDs) represent the leading cause of mortality worldwide, with 20.5 million deaths in 2022 [5]. However, the causal relationship between ECs and both the development and progression of CVDs remains elusive. Thus, there is limited evidence of the mechanisms by which EC exposure harms the cardiovascular system.

The basic elements of the cardiovascular system are excitable cells, including cardiomyocytes, pacemaker cells, conducting cells, and vascular smooth muscle, which—when connected in a network—form the heart and various vessels to maintain optimal cardiovascular function [6]. A key characteristic of excitable cells is that they are rich in membrane-embedded ion channels that are selective to a wide array of ions. These channels, which link the intracellular and extracellular environments, are essential for various physiological processes, including membrane excitability, synaptic transmission, signaling, and cell volume regulation [7]. The coordinated conduction of excitatory stimuli, which results in the generation of action potentials in cardiomyocytes via the opening and closing of ion channels, is crucial for maintaining normal cardiac activity [8]. Ion channels are therefore important and attractive targets for drug development in diseases such as diabetes, epilepsy, hypertension, cancer, and chronic pain [9]. ECs and drugs specifically designed to target ion channels are complex compounds that may share similarities in chemical structure, functional groups, molecular weight, polarity, biological activity, toxicity mechanism, metabolism, and environmental behavior. These similarities could mean that ECs inadvertently affect the functioning of certain ion channels [10]. A growing number of studies indicate that pollutants can significantly disrupt ion channels, leading to alterations in action potential dynamics, calcium homeostasis, and reactive oxygen species (ROS) balance [11,12]. However, a comprehensive understanding of how different ECs interfere with cardiac ion channel function, along with the potential synergistic effects between various ECs, remains limited. Addressing this knowledge gap is crucial for assessing the cardiovascular risks associated with EC exposure and developing effective strategies to mitigate any negative impacts.

Currently available research on the health effects of ECs reveals limited progress in the field of electrophysiology, primarily due to several factors: the complexity of toxic mechanisms, high technical demands, insufficient interdisciplinary collaboration, substantial research costs, data interpretation challenges, and divergent research priorities. However, future research approaches could be promising due to technological advancements and enhanced interdisciplinary cooperation. The purpose of this review is to provide an overview of current knowledge regarding the influence of ECs on cardiac ion channels. To this end, we will systematically discuss the major ion channels, along with their associated functions in the cardiovascular system. Furthermore, we will focus on the link between exposure to nine typical ECs and the adverse effects on ion channels, including the possible underlying mechanisms.

## 2. Literature Review and Methodology

A narrative literature review was conducted for full-text original articles and reviews from PubMed, Web of Science, and Google Scholar databases, covering literature published up to March 2026.

The search strategy employed keywords including “emerging contaminants” OR “per- and polyfluoroalkyl substances” OR “PFAS” OR “bisphenol A” OR “BPA” OR “flame retardants” OR “microplastics” OR “phthalates” OR “polycyclic aromatic hydrocarbons” OR “PAHs” OR “polychlorinated biphenyls” OR “PCBs” OR “pesticides” OR “pharmaceutical residues” in conjunction with “cardiac ion channels” OR “cardiovascular toxicity” OR “heart ion channels” OR “cardiotoxicity” OR “arrhythmias” OR “cardiovascular diseases” OR “CVD”. Refinements were applied to focus on ion channel-specific mechanisms (e.g., adding “sodium channels” OR “calcium channels” OR “potassium channels”), reducing the number to 519 articles, and further to clinical and epidemiological evidence by incorporating “epidemiology” OR “clinical study” OR “cohort” OR “human exposure,” resulting in 73 articles. The detailed flowchart of literature selection is provided in Supplementary Figure S1.

Eligible studies included original research articles as well as relevant review articles providing mechanistic or translational insights. Only articles published in English with full-text availability were considered. Studies were included if they examined exposure to at least one of the targeted contaminant classes and reported cardiac electrophysiological endpoints, ion channel function or expression, intracellular calcium dynamics, or related cardiovascular outcomes. Studies conducted in non-cardiac tissues were included where they provided mechanistic insights not yet available from cardiac models, with their tissue origin explicitly noted. Non-peer-reviewed reports, conference abstracts without full-text availability, and studies lacking sufficient methodological detail were excluded.

## 3. The Adverse Effects of Emerging Contaminant Exposure on Cardiovascular Ion Channels: Evidence from In Vivo and In Vitro Studies

The growing prevalence of ECs in environmental and biological systems is now a public health concern, mainly because these compounds are persistent and have the capacity to disrupt fundamental physiological processes [13]. Among the myriad toxicological effects, these pollutants can interfere with integral membrane proteins, including ion channels, that govern electrical signaling, cellular communication, and homeostasis. Cardiac ion channels are fundamental to the electrical and contractile activities of the heart, as these channels orchestrate the precise regulation essential for normal cardiac physiology. These membrane proteins, which comprise sodium, calcium, potassium, chloride, and non-selective cation channels, govern action potential generation, propagation, and repolarization to ensure coordinated cardiac function (Text S1, Table S1). For these reasons, they are vulnerable targets for pollutant-induced dysregulation.

The following sections examine the deleterious effects of various ECs—including PFAS, BPA, and FRs, as well as MPs, PAEs, PAHs, PCBs, pesticides, PPCPs—on cardiac ion channel function. These substances, which are ubiquitous in environmental media and human tissues, infiltrate biological systems through dietary, respiratory, and dermal routes [14]. The structural diversity of ECs enables interactions with ion channels via multiple pathways, which include direct binding to channel proteins, modulation of gene expression, induction of oxidative stress, epigenetic alterations, and disruption of calcium homeostasis. By examining alterations in sodium, calcium, potassium, and other ion channels, as well as the associated molecular pathways, this section highlights the potential mechanisms by which these pollutants compromise cardiac health, ultimately contributing to arrhythmias, contractile dysfunction, and other cardiovascular diseases. Effects of typical emerging contaminant exposure on cardiac ion channels are summarized in Table 1. To facilitate interpretation of the strength and relevance of the summarized evidence, each study was further classified into three evidence levels according to the availability of direct electrophysiological recordings, cardiac model-based molecular or functional evidence, and indirect supportive findings. The evidence levels (A, B, and C) assigned to each study in the table are defined as follows: A = Direct electrophysiological evidence: Ion currents (e.g., *I*_Na_, *I*_Ca_, *I*_Kr_) or action potential parameters (dV/dtmax, APD) were directly recorded via patch-clamp in cardiomyocytes or cardiac tissue, or intracellular Ca^2+^ transients were measured by calcium imaging; B = Indirect strong evidence: Significant changes in cardiac ion channel protein or mRNA expression, or whole-heart electrophysiological phenotypes (e.g., ECG abnormalities, arrhythmia susceptibility) were observed in cardiac models, but direct current recordings were not performed; C = Indirect supportive evidence: Findings are derived from developmental models (e.g., zebrafish embryos), non-cardiac tissues (e.g., vascular smooth muscle, neurons), or transcriptomic screening without direct electrophysiological validation in mature cardiomyocytes.

### 3.1. Definition and Scope of “Ion Channels” in This Review

Cardiac ion channels are fundamental to the electrical and contractile activities of the heart, as these channels orchestrate the precise regulation that is essential for normal cardiac physiology. Cardiac ion channels, encompassing sodium, calcium, potassium, chloride, and non-selective cation channels, play a critical role in regulating the generation, propagation, and repolarization of action potentials, which is essential for maintaining coordinated cardiac function [25]. In this study, we adopt a broad conceptualization of ion channels, with the term not only describing ion channels but also any related pumps and exchangers. For instance, voltage-gated sodium channels and the associated Na^+^/Ca^2+^ exchanger 1 (NCX1) initiate depolarization and help maintain calcium homeostasis, respectively, while calcium channels, including L-type voltage-gated Ca^2+^ channels and ryanodine receptors (RyRs), drive contraction and signaling [26]. Potassium channels, the most diverse group of ion channels, stabilize membrane potential and repolarization, with subtypes like the voltage-gated K^+^ channel and inwardly rectifying K^+^ channel playing distinct roles in the maintenance of heart rhythm [27]. Chloride and transient receptor potential (TRP) channels further modulate excitability and cellular homeostasis, thereby contributing to heart resilience [28]. The dysregulation of these channels is implicated in arrhythmia, heart failure, and hypertrophy, which highlights a critical role in cardiac health. A summary of the various types of cardiac ion channels, categorized by the preferred ion, is a prerequisite to comprehensively understanding the interactions between ECs and cardiac ion channels.

### 3.2. Emerging Contaminants Disrupt Cardiovascular Calcium Channels and Intracellular Calcium Homeostasis

Calcium channels, particularly L-type voltage-gated calcium channels (LTCCs) and intracellular calcium-release channels like ryanodine receptors (RyRs) and inositol 1,4,5-trisphosphate receptors (IP_3_Rs), are the most extensively documented targets for EC-induced cardiotoxicity. LTCCs are central to excitation–contraction coupling, and their inhibition or abnormal activation directly impairs the cardiovascular system.

**Figure 1:**
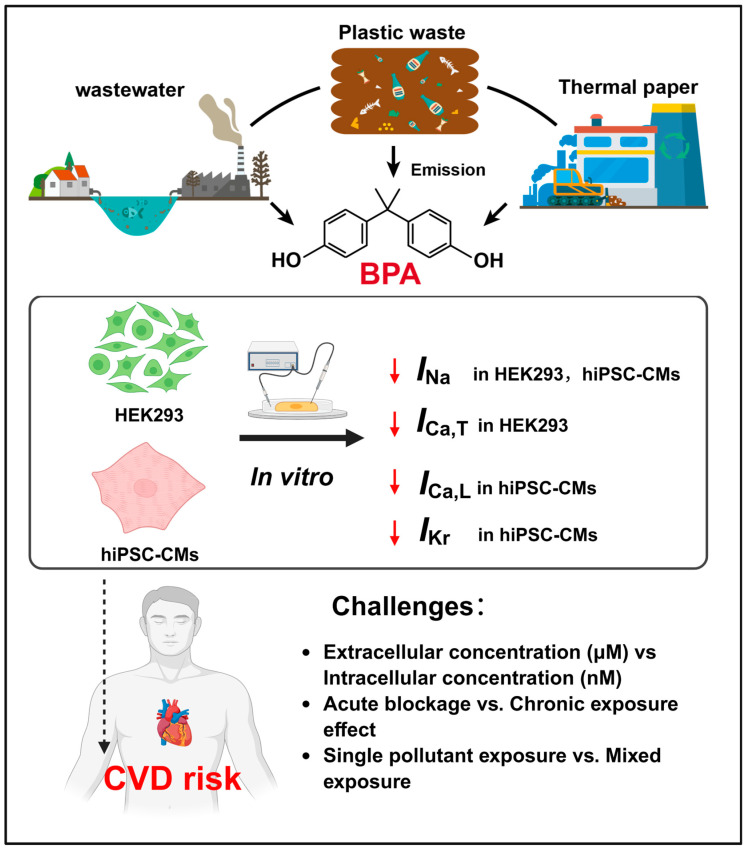
The effects of BPA on ion channels in cardiac models and verification of the challenge posed by BPA exposure to the risk of cardiovascular diseases in humans. The red downward arrow represents inhibition.

LTCCs are particularly susceptible to inhibition and functional interference by both therapeutic drugs and exogenous substances, and represent major pharmacological targets in the treatment of cardiovascular diseases. Owing to the structural diversity of emerging contaminants, they can interact with LTCCs through multiple direct and indirect mechanisms. Acute exposure experiments on cardiac models have found that BPA can inhibit calcium current, suggesting a possible risk of cardiovascular disease (Figure 1). Deutschmann et al. reported that BPA (1–100 μM) rapidly inhibits calcium currents through L- and T-type Ca^2+^ channels in rat cardiomyocytes [29]. Moreover, experiments in which zebrafish embryos were subjected to chronic BPA exposure (25 μM) demonstrated a downregulation of LTCC mRNA and sarcoplasmic/endoplasmic reticulum calcium ATPase (SERCA) expression, leading to reduced embryonic heart rate and cardiac contraction amplitude; these results further support the link between calcium homeostasis disruption and cardiac dysfunction [30].

Exposure to PAHs, such as phenanthrene, leads to a dose-dependent reduction in beat cycle and field potential duration in human induced pluripotent stem cell-derived cardiomyocytes (hiPSC-CMs) primarily through LTCC inhibition [31]. Recent studies further demonstrate that long-term low-dose exposure to polystyrene microplastics and nanoplastics (PS-MNPs) significantly impairs contractility, reduces Ca^2+^ transient amplitude, and alters Ca^2+^ handling dynamics, accompanied by mitochondrial dysfunction and pro-hypertrophic effects even at environmentally relevant concentrations [32]. Phthalates also exhibit clear cardiotoxic potential in hiPSC-CM models, disrupting calcium homeostasis and ion channel function [33].

Positively charged PS-MNPs are rapidly internalized by neonatal rat cardiomyocytes, leading to the acute inhibition of LTCCs, a significant reduction in intracellular Ca^2+^ levels, and decreased contractility [34]. PAEs like diethyl phthalate (DEP) have been shown to suppress Ca^2+^ currents and downregulate Cav1.2 mRNA expression in rat aortas, causing abnormal vascular relaxation [35]. Certain PPCPs, such as the antihypertensive drug amlodipine, are specifically designed to block the α1 subunit of Cav1.2. However, excessive environmental exposure to such pharmaceutical residues may inadvertently suppress myocardial contractility in non-target organisms [36].

**Figure 2:**
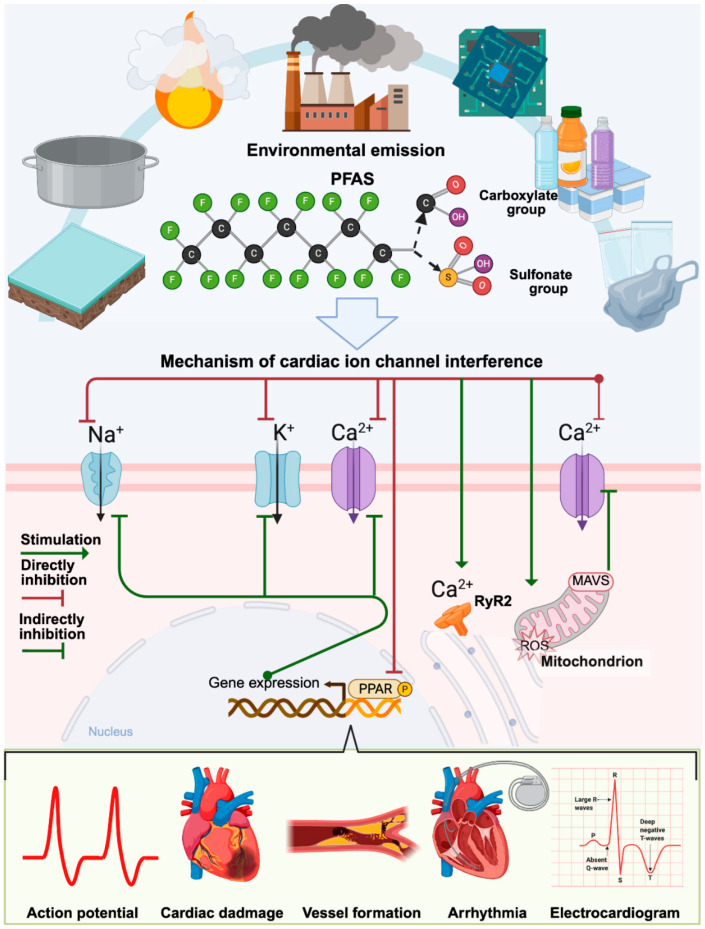
Schematic overview of PFAS environmental emissions, structures, and proposed mechanisms of cardiac ion channel interference leading to cardiovascular damage. PFAS may affect Na^+^, K^+^, and Ca^2+^ channels through direct binding, oxidative stress, Ca^2+^ release (RyR2), and nuclear receptor (PPAR) modulation affecting gene expression.

Conversely, some ECs induce intracellular Ca^2+^ overload. In recent years, several studies have reported that PFAS, such as PFOA and PFOS, can interact with ion channels to yield potentially disruptive effects (Figure 2). PFAS exposure has been linked to increased intracellular Ca^2+^ concentrations in primary chicken embryo cardiomyocytes, alongside decreased cell viability [37]. The mitochondrial toxicity of PFOS exposure observed in cardiomyocytes derived from embryonic stem cells was found to inhibit in vitro myocardial formation, reduce L-type Ca^2+^ channel expression, and block the transient increase in [Ca^2+^] in cardiomyocytes exposed to 100 μM PFOS [38].

Feiteiro et al. utilized A7r5 vascular smooth muscle cells and patch-clamp techniques to show that TBBPA inhibits LTCCs to decrease Ca^2+^ influx. Although this confirms LTCC susceptibility in excitable cells, direct evidence in cardiomyocytes is required to establish cardiac relevance. Additionally, the researchers hypothesized that the vascular effects of TBBPA may be mediated by the activation of α1-, β1-, and β2-adrenoceptors, in concert with its actions on Ca^2+^ and K^+^ channels [39].

PAEs like di(2-ethylhexyl) phthalate (DEHP) also trigger Ca^2+^ overload, activating the CaMKII-RIPK3 necroptosis pathway in cardiomyocytes [40]. In mouse embryonic stem cell-derived cardiomyocytes, the commercial PCB mixture Aroclor 1254 inhibited spontaneous Ca^2+^ oscillations, reduced sarcoplasmic reticulum Ca^2+^ content, and suppressed voltage-gated Ca^2+^ entry, leading to impaired contractile function [41]. Despite a concurrent upregulation of RyR2 mRNA (1.7-fold), the net functional effect was suppression of intracellular Ca^2+^ dynamics, suggesting that the transcriptional response may reflect a compensatory mechanism rather than direct channel activation.

Pesticides, such as pyrethroids, can enhance the permeability of voltage-gated Ca^2+^ channels, causing calcium signaling abnormalities that have been primarily characterized in neuronal and insect models [42]. Whether these calcium-handling defects propagate to human cardiomyocyte excitation–contraction coupling remains to be investigated. Additionally, non-pharmaceutical PPCPs like the antimicrobial agent triclosan can elevate intracellular Ca^2+^ levels within rat smooth muscle cells and reduce mitochondrial membrane potential, leading to vascular endothelial dysfunction [43]. Emerging organophosphate flame retardants (2-ethylhexyl diphenyl phosphate and diphenyl phosphate) also induce differential cardiotoxicity in zebrafish models through disruption of calcium homeostasis and related signaling pathways [44].

In summary, L-type calcium channels emerge as the most consistently affected target across ECs, with evidence ranging from direct current blockade (BPA, PAHs) to disruption of accessory calcium-handling proteins (PFAS, PAEs, FRs, MPs, PCBs). T-type calcium channels and intracellular release channels remain comparatively understudied in the context of EC cardiotoxicity.

### 3.3. Emerging Contaminants Interfere with Cardiovascular Voltage-Gated Sodium Channels

Voltage-gated sodium channels, notably Na_V_1.5, are responsible for phase 0 depolarization of the cardiac action potential and are essential for maintaining conduction velocity. Disruption of these channels by ECs can lead to conduction blocks and ventricular arrhythmias.

Beyond direct binding, BPA can indirectly affect electrophysiological function by regulating the expression of ion channel-related genes or the activity of encoded proteins. Experiments in hiPSC-CMs revealed that BPA exerts dose-dependent inhibition of the sodium channels to impair calcium flows and contractile homeostasis [23]. In HEK-transfected cell lines, BPA directly binds to the pore domain of Na_V_1.5, causing a dose-dependent blockade of sodium currents [45]. A recent study using whole-cell voltage-clamp recordings confirmed that BPA is a potent inhibitor of both peak sodium current (*I*_NaP_) and late sodium current (*I*_NaL_) in Na_V_1.5-transfected cells, with IC_50_ values of 55.3 μM and 23.6 μM, respectively; BPA and its analogue BPF also impaired atrioventricular conduction in whole-heart preparations [46].

This direct binding mechanism is mirrored by PAE metabolites; for example, mono-(2-ethylhexyl) phthalate (MEHP) inhibits Na_V_1.5 in isolated rat hearts, which significantly slows epicardial conduction velocity [47]. Additionally, in hiPSC-CMs, PFOA has been shown to inhibit *I*_Na_ and downregulate SCN5A mRNA [22]. Pharmaceutical residues also contribute to this blockade; for example, the widely prescribed antidepressants citalopram and escitalopram are frequently detected in global surface waters and directly inhibit human Na_V_1.5 channels expressed in HEK293 cells, reducing peak *I*_Na_ by approximately 60% and shifting the inactivation curve toward more negative potentials [48].

While most of the aforementioned ECs inhibit sodium currents, exposure to certain PAHs and pesticides has been reported to increase sodium influx. In trout ventricular cardiomyocytes, phenanthrene and retene increase *I*_Na_, creating an imbalance between sodium and potassium currents that induces electrophysiological instability [49]. Pyrethroid insecticides bind to voltage-gated sodium channels and delay their inactivation, maintaining a persistent sodium influx [50]. A systematic review and meta-analysis confirmed that pyrethroid-induced *I*_NaL_ enhancement is a key mechanism underlying action potential prolongation, QT interval extension, and impaired cardiac contractility [51].

Organochlorine pesticides also target sodium channels by prolonging the channel opening time, a mechanism primarily characterized in neuronal preparations. However, studies have shown that at nanomolar concentrations, Aldrin can directly act on the sodium channels of cardiomyocytes (such as Na_V_1.5), interfering with the electrophysiological activities of cardiomyocytes [52].

### 3.4. Emerging Contaminants Modulate Cardiovascular Potassium Channels and Repolarization

Potassium channels, including the rapid delayed rectifier potassium current (*I*_Kr_/hERG), dictate myocardial repolarization and action potential duration (APD). Inhibition of these channels prolongs the APD, elevating the risk of QT interval prolongation and lethal arrhythmias such as torsades de pointes.

PAHs are potent disruptors of potassium channels. In heterologous expression systems, phenanthrene inhibits *I*_Kr_, causing calcium cycling disorders and prolonging action potentials; this toxic effect is notably amplified in the presence of hERG channel mutations [53]. Marris et al. (2025) reported in female sheep ventricular myocytes that phenanthrene induced a paradoxical shortening of APD90 (~37%) despite substantial *I*_Kr_ inhibition (~56% at 25 μM) [12]. This unexpected effect was primarily attributed to the dominant suppression of calcium handling, particularly via inhibition of SERCA and RyR2, which overrides the canonical APD-prolonging consequence of *I*_Kr_ blockade, thereby highlighting the complex multi-channel interactions of PAHs.

BPA also exhibits strong inhibitory effects on *I*_Kr_. Ma et al. demonstrated that acute BPA exposure rapidly inhibits *I*_Kr_ in female canine ventricular myocytes, leading to proarrhythmic phenotypes like early afterdepolarizations [54]. Pharmaceutical residues, particularly Class III antiarrhythmics (e.g., amiodarone, sotalol), directly block Kv11.1 (the *I*_Kr_ channel), delaying repolarization and prolonging the QT interval [55]. Lee et al. (2025) demonstrated that di-n-octyl phthalate (DnOP) markedly prolonged field potential duration in hiPSC-CMs by inhibiting *I*_Kr_, posing a stronger proarrhythmic risk than DEHP, whereas dibutyl phthalate (DBP) and benzyl butyl phthalate (BBP) primarily induced beating arrest with decreased amplitude rather than repolarization abnormalities [33].

In contrast, some flame retardants act as potassium channel activators. In the A7r5 vascular smooth muscle cell line, TBBPA increased mRNA levels of BK_Ca_1.1α- and β1-subunits, which also suggests that K^+^ channel activation may represent another pathway by which TBBPA mediates vascular relaxation [39]. Moreover, Liu et al. found that natural polybrominated diphenyl ethers selectively activate the Kv7.1 and Kv7.2 potassium channels, which suggests that these compounds are potential potassium channel activators [56]. This bidirectional disruption (inhibition by BPA/PAHs/PPCPs in cardiac models vs. activation by FRs in vascular smooth muscle) highlights the complex, chemical-specific nature of EC interference with cardiac ion channels. Whether FRs similarly modulate cardiac potassium currents remains to be established.

### 3.5. Emerging Contaminants Impair Cardiovascular Ion Transporters and Exchangers

Beyond pore-forming ion channels, ECs severely impact the ion transporters and ATPases required to maintain transmembrane ionic gradients. Dimitriadi et al. observed that zebrafish exposed to sublethal concentrations of PS-MNPs exhibited decreased heart rates and reduced ventricular contraction frequency [57]. Mechanistic studies further revealed that PS-MNPs may bind to β-adrenergic receptors (key receptors regulating heart rate) in cardiomyocytes to inhibit downstream signaling. On the other hand, PS-MNPs can also interact with Na^+^/K^+^ ATPases (NKA), which are critical to maintaining myocardial ion gradients, to disrupt the Na^+^/K^+^ balance and cause abnormal action potential. Research by Hong et al., which relied on *Gobiocypris rarus* as a model organism, found that 96 h exposure to triphenyl phosphate (TPHP) and tris(1,3-dichloro-2-propyl) phosphate (TDCIPP) reduces embryonic heart rate. Mechanistic studies further revealed that both compounds inhibit Na^+^/Ca^2+^ exchangers, disrupting Na^+^ and Ca^2+^ transport and potentially leading to myocardial contractile dysfunction and arrhythmias [58]. In another study, exposure to tri-o-cresyl phosphate (ToCP) and tri-m-cresyl phosphate (TmCP) impaired overall ion and osmoregulatory functions in zebrafish; these changes were attributed to reduced Ca^2+^-ATPase and Na^+^/K^+^-ATPase activities, along with decreased ATP and K^+^ levels [59]. In H9C2 rat cardiomyocyte, hexabromocyclododecane (HBCD) exposure disrupts the expression balance of RyR2, SERCA2a, and NCX1, leading to sarcoplasmic reticulum Ca^2+^ overload and impaired Ca^2+^ reuptake during cardiac relaxation [19]. Additional experiments utilizing mouse cardiomyocytes demonstrated that DBP exposure induces endoplasmic reticulum stress and mitochondrial damage, both of which reduce mitochondrial membrane potential. The underlying mechanism involves disruption of the calcium transfer pathway mediated by inositol 1,4,5-trisphosphate receptor type 1 (IP_3_R1, endoplasmic reticulum release channel), glucose-regulated protein 75 (GRP75, linker protein), and the voltage-dependent anion-selective channel 1 (VDAC1, mitochondrial calcium channel) [60]. However, this field requires further attention, as there is a lack of direct evidence on the association between PAE exposure and chloride and TRP channel effects despite the evidence described above. Novel insecticides target insect-specific TRP channels (e.g., TRPA1/TRPV) to achieve precise lethality through the activation of nociceptive pathways. However, since these channels are also expressed in cardiomyocytes, careful evaluation of potential cross-reactivity risks is needed [61].

## 4. Clinical and Epidemiological Evidence of Cardiovascular Diseases Induced by Emerging Contaminant Exposure

Recent human studies have begun to link exposure to various emerging contaminants with measurable cardiac electrical changes and clinical CVD endpoints (Table 2). These findings provide hypothesis-generating clues, but they do not directly measure ion channel activity or establish exact causal mechanisms. The evidence strength of individual studies was assessed as follows: Moderate evidence is assigned to studies with direct ECG or biomarker endpoints and adequate sample size (*n* > 1000), though cross-sectional in design; Low evidence is assigned to studies with indirect endpoints (e.g., self-reported disease, atherothrombotic events) or small sample size (*n* < 100).

For example, a 2023 cross-sectional study of 1229 middle-aged Chinese adults found that higher serum PFAS levels were associated with slower atrioventricular conduction (longer PR interval) and shorter QRS duration on electrocardiogram (ECG), as well as reduced heart rate [62]. A more recent cross-sectional study of 3450 older adults (≥60 years) further strengthened this link, showing that higher serum concentrations of multiple PFAS were associated with greater odds of both minor and major ECG abnormalities, including prolonged QRS duration and QTc interval, as well as shortened T-wave duration [63].

In a large US cohort (the Fernald Community Cohort), higher urinary BPA and bisphenol F (BPF) were significantly associated with PR interval and QRS complex prolongation in women and corrected QT interval (QTc) prolongation (associated with triclocarban) in men [64].

In the National Health and Nutrition Examination Survey (NHANES) (7032 US adults), higher serum polybrominated biphenyl-153 and related brominated flame retardant levels were linked to markedly increased odds of heart failure and coronary heart disease [65]. PAE exposures have also been linked to cardiac injury in humans; in NHANES (n ≈ 1237 adult men), higher urinary PAE metabolites (e.g., mono(3-carboxypropyl) phthalate, mono-isobutyl phthalate, and mono-benzyl phthalate) were positively correlated with elevated serum troponin I/T levels [66].

High PAH exposure, as measured by urinary hydroxylated metabolites, has been associated with greater CVD prevalence in US adults [67]; for instance, individuals in the highest quartile of 2-OH-fluorene had approximately 50–100% higher odds of self-reported heart disease. Emerging evidence has additionally highlighted MNPs as novel contributors to cardiovascular risk. In a prospective multicenter study of patients undergoing carotid endarterectomy, the presence of MNPs (particularly polyethylene and polyvinyl chloride) within atherosclerotic plaques was associated with a markedly higher risk of the composite endpoint of myocardial infarction, stroke, or all-cause death (hazard ratio 4.53) during follow-up [68]. Scoping reviews and additional human tissue analyses have detected MNPs in atherosclerotic plaques, thrombi, and cardiac tissues, supporting their potential role in promoting inflammation, oxidative stress, and plaque instability [69,70].

In summary, exposure to multiple ECs is significantly associated with alterations in human ECG parameters (including PR, QRS, and QTc interval prolongation, as well as abnormal heart rate) and increased cardiovascular disease risk. These clinical observations are broadly compatible with the cardiotoxic mechanisms identified in experimental models and provide hypothesis-generating evidence that merits further investigation. However, the epidemiological findings represent statistical associations rather than demonstrations of specific causal pathways. Prospective cohort studies that integrate exposure biomarkers with high-resolution ECG phenotyping will be essential to further clarify the nature and magnitude of cardiovascular risks associated with emerging contaminant exposure in human populations.

**Table 2:** Effects of emerging contaminants on cardiovascular diseases: evidence from clinical and epidemiological studies.

Contaminant	Cohort	Key Findings	Evidence Strength	Ref.
PFASs	Chinese adults, *N* = 1229; mean age ≈ 55	↑PR, ↓QRS; ↓HR	Moderate	[62]
Phthalates	US adults, *N* ≈ 1237; ≥20 years	↑Troponin I/T elevation	Moderate	[66]
Bisphenols	US adults, *N* ≈ 1000; 18–80 years	↑PR, ↑QRS, QTc prolongation;	Moderate	[64]
Brominated flame retardants	US adults, *N* = 7032; ≥20 years	Heart failure, coronary heart disease	Low	[65]
Polycyclic aromatic hydrocarbons	US adults, *N* = 9136; 20–79 years	Coronary/ischemic heart disease, stroke	Moderate	[67]
Pesticides	Myanmar adults, *N* = 90; 20–40 years	QTc prolongation and increased CVD risk	Low	[71]
Microplastics and nanoplastics	Patients, *N* = 304; 18–75 years	Increased risk of subsequent cardiovascular events	Low	[68]

↑, the value was significantly higher than the control group; ↓, the value was significantly lower than the control group. Abbreviations: AV, atrioventricular; CVD, cardiovascular disease; HR, heart rate; PFAS, per- and polyfluoroalkyl substances; PR, PR interval; QRS, QRS duration; QT, QT interval; QTc, corrected QT interval.

## 5. Conclusions and Further Perspectives

**Figure 3:**
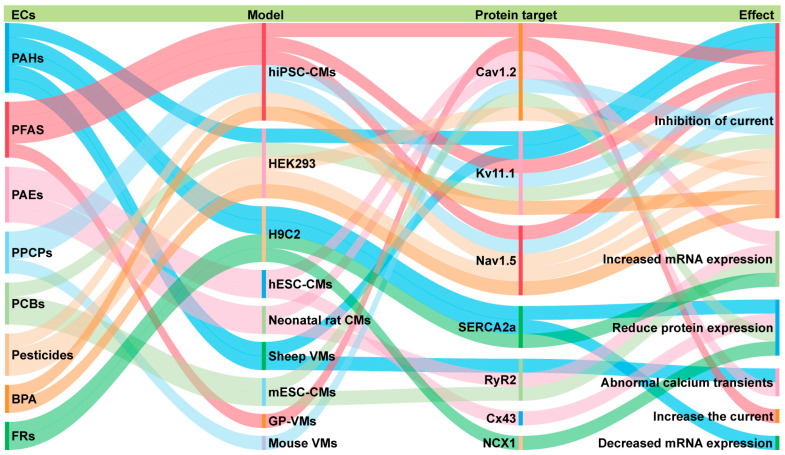
Schematic overview of the effects of ECs on cardiac ion channels, transporters, and calcium-handling proteins across cardiac models. Abbreviations: CMs, cardiomyocytes; VMs, ventricular myocytes; GP, guinea pig; hiPSC, human induced pluripotent stem cells; hESC, human embryonic stem cells; mESC, mouse embryonic stem cells.

The reviewed literature collectively indicates that multiple classes of ECs can disrupt cardiac excitability by targeting ion channels, transporters, and calcium-handling proteins. Figure 3 illustrates the effects of ECs on cardiac ion channels, transporters, and calcium-handling proteins across cardiac models. Clear patterns emerge across the different classes of contaminants investigated. BPA, PFAS, certain pesticides, and PPCPs exhibit a relatively broad ion channel-targeting profile, with direct evidence for modulation of Nav1.5, Cav1.2, and Kv11.1 currents in cardiac models. PAHs, particularly phenanthrene, primarily target Kv11.1 and additionally affect calcium transients and the expression of calcium-cycling proteins. By contrast, PAEs, FRs, and PCBs show limited direct ion channel effects; the predominant findings for these classes involve altered calcium-cycling protein and mRNA expression. For MNPs, studies in cardiac models remain particularly scarce. These interactions have the potential to impair cardiac function, as reflected in experimental phenotypes such as arrhythmia, bradycardia, and disrupted calcium handling. However, the translation of this mechanistic insight to human cardiovascular risk remains constrained by the evidence gaps and model limitations.

Translating the above mechanistic and epidemiological evidence into human cardiovascular risk assessment requires consideration of two interrelated factors: the substantial discrepancy between experimental concentrations and actual internal exposure levels in humans, and the inherent limitations of model systems for extrapolation. For several major contaminant classes, the micromolar concentrations used in most patch-clamp and calcium-imaging studies far exceed those measured in the general population. Median serum levels of PFOA (~3 nM) are one and a half to four and a half orders of magnitude below the 0.1–100 μM concentrations used in hiPSC-CM studies [72], while serum BPA concentrations (~8 nM) are 100- to 10,000-fold lower than typical experimental concentrations [73]. Although these differences are valuable for hazard identification, they highlight several understudied factors such as tissue accumulation, metabolite toxicity, and cumulative effects of chronic low-dose exposure. Moreover, variations across rodent, zebrafish, hiPSC-CM, and heterologous expression models, together with the heart’s compensatory capacity and chronic structural remodeling, limit definitive conclusions about human cardiac risk. The case of micro- and nanoplastics (MNPs) deserves particular caution, as their detection in atherosclerotic plaques and cardiac tissue is associated with adverse cardiovascular events, yet direct electrophysiological evidence of ion channel dysfunction remains limited and largely inferential.

Based on prior research findings, future investigations should prioritize the following three key areas:

(1) Establish large-scale cohort studies with long-term follow-up periods.

Future research should integrate environmental exposure databases and cardiovascular epidemiological data to establish long-term cohort studies. Investigating the causal relationship between pollutant exposure and cardiac electrophysiological abnormalities (e.g., arrhythmia, QT prolongation, conduction block) will be essential. In addition, wearable devices (e.g., Holter monitors) can provide continuous cardiac activity data for more precise assessments.

(2) Elucidate ion channel modulation mechanisms.

Advanced techniques such as patch-clamp electrophysiology, optogenetics, and high-resolution calcium imaging should be used to examine how emerging contaminants alter ion channel kinetics (e.g., activation, inactivation, and recovery time constants) and how these mechanisms are involved in arrhythmogenesis.

(3) Develop interventions to mitigate EC-induced channelopathies.

To address the cardiovascular risks posed by ECs, future research should focus on developing practical interventions to mitigate EC-induced ion channel dysfunction. High-throughput screening of small molecules that protect or restore cardiac ion channel function offers a promising approach to identifying novel therapeutics, drawing parallels from antiarrhythmic drug development. Additionally, exploring nutritional and pharmacological interventions, such as antioxidants to counteract oxidative stress or calcium-stabilizing supplements to restore ionic homeostasis, could provide effective strategies to protect exposed populations and reduce the incidence of pollutant-related cardiac electrophysiological abnormalities.

## Figures and Tables

**Table 1:** Effects of typical emerging contaminant exposure on cardiac ion channels.

Model Type	Model	Pollution	Major Findings	Interpretation	Evidence Level	Ref.
In vivo	Sprague-Dawley rats	PFOS; 20 mg/kg/day; 4 weeks	↓Cx43, ↓SERCA2	Exposure to PFOS decreased Cx43 and SERCA2 expression, impairing intercellular electrical coupling and SR Ca^2+^ handling.	B	[15]
In vivo	Wistar rats	Chlordecone; 0–1 µg/L; 4 weeks	↓*Kcnq1*, ↓*Scn5a*, ↓Cx43, ↓Cx40	Exposure to chlordecone decreased *Kcnq1*, *Scn5a*, Cx43, and Cx40 expression, promoted atrial electrical remodeling, and increased vulnerability to atrial fibrillation.	B	[16]
In vivo	Wistar rats	Rotenone; 1.0 mg/kg/d; 2 weeks	↓Cx43, ↓KCNH2; ↑Kir2.1, ↑Kir6.2, ↑Cav1.2	Exposure to rotenone decreased Cx43 and KCNH2 but increased Kir2.1, Kir6.2, and Cav1.2 expression, producing cardiac electrical remodeling that may favor arrhythmogenesis.	B	[17]
In vivo	C57BL/6n mice	BPA; 200 µg/kg/day; Lifelong	↑SERCA2a, ↑NCX1	Lifelong exposure to BPA increased SERCA2a and NCX1 expression, indicating altered intracellular Ca^2+^ handling.	B	[18]
In vivo	Zebrafish	HBCD; 0–200 nM; 72 hpf	↓*KCND2*	Exposure to HBCD reduced *KCND2* expression through the miR-1/Irx5 pathway, disrupted K^+^ homeostasis, and contributed to arrhythmogenic remodeling.	C	[19]
In vivo	Zebrafish	Phenanthrene; 0.05–50 nM; 72 hpf	↓SERCA2	Exposure to phenanthrene decreased SERCA2 expression via the TBX5-SERCA2a pathway, impaired Ca^2+^ handling, and increased susceptibility to arrhythmia.	C	[20]
In vivo	*Oncorhynchus mykiss*	Retene; 32 μg/L; 3 days	↑*cacng8b*, ↓*atp2a1l*, ↓*slc24a2*, ↓*atp1a1a.1*, ↓*atp1a1a.4*, ↓*atp1b1b*, ↓*kcnj1a.1*, ↓*kcnc3a*	Exposure to retene altered the expression of multiple ion transport- and Ca^2+^-handling-related genes, indicating disruption of cardiac electrophysiological homeostasis.	C	[21]
In vitro	hiPSC-CMs	PFOA; 0.1–100 μM; 24 h	↓ dV/dtmax, ↓APD; ↓*I*_Na_, ↓*I*_Ca_, ↓*I*_Kr_; ↓*SCN5A*, ↓*CACNA1C*, ↓*KCND3*, ↓*KCNH2*, ↓*KCNQ1*, ↓*KCNJ2*	Exposure to PFOA reduced the function and expression of multiple cardiac ion channels, attenuated action potential upstroke and duration, and impaired cardiomyocyte electrophysiology.	A	[22]
In vitro	hiPSC-CMs	BPA; 1–100 μM; 24 h	↓dV/dtmax, ↓APD50, ↓APD90; ↓*I*_Na_, ↓*I*_Ca_, ↓*I*_Kr_;	BPA disrupts calcium transients and cardiac contraction by inhibiting multiple cardiac ion channels.	A	[23]
In vitro	hESC-H9	TBBPA; 1–100 nM; 8 days	↓Cardiomyocyte beating; ↓*TNNT2*	Exposure to TBBPA reduced *TNNT2* expression and beating activity, indicating impaired cardiomyocyte maturation and functional development.	B	[24]
In vitro	H9C2	HBCD; 24 h	↓NCX1; ↑Serca2a; ↑RyR2;	Exposure to HBCD decreased NCX1 but increased Serca2a and RyR2 expression, suggesting disturbed Ca^2+^ homeostasis that may contribute to rhythm abnormalities.	B	[19]

↑, the value was significantly higher than the control group; ↓, the value was significantly lower than the control group. Abbreviations: APD, action potential duration; BPA, bisphenol A; casq2, calsequestrin 2; CV, conduction velocity; Cx40, connexin 40; Cx43, connexin 43; dV/dtmax: maximal upstroke velocity; hpf, hours post-fertilization; HBCD, hexabromocyclododecane; hiPSC-CMs, human induced pluripotent stem cell-derived cardiomyocytes; Irx5, Iroquois homeobox 5; miR-1, microRNA-1; NCX1, Na^+^/Ca^2+^ exchanger 1; PFOS, perfluorooctane sulfonate; SR, sarcoplasmic reticulum; TBX5, T-box transcription factor 5. Evidence level definitions: A = Direct electrophysiological evidence; B = Indirect strong evidence; C = Indirect supportive evidence.

## Data Availability

The data presented in this study are available upon request from the corresponding author.

## Supplementary Materials

The following supporting information can be downloaded at: https://www.mdpi.com/article/10.3390/toxics14050450/s1. Text S1: Major ion channels of the cardiovascular system [74,75,76,77,78,79,80,81,82,83,84,85,86,87,88,89,90,91,92,93,94,95,96,97,98,99,100,101,102,103,104,105,106,107,108,109,110,111,112]; Table S1: Ion Channels in Cardiac Cells: Types, Functions, and Associated Cell Types; Figure S1: A flow diagram for the narrative literature review.
