# Supplementary material for: Emerging Environmental Contaminants Targeting Cardiovascular Ion Channels: Exposure Effects, Underlying Mechanisms, and Implications for Cardiovascular Health Risks
Source: Toxics. 2026 May 21;14(5):450. doi: 10.3390/toxics14050450 (PMC13211365; doi:10.3390/toxics14050450)
Supplement: Supplementary file 1 [file toxics-14-00450-s001.zip › toxics-4289993-supplementary.pdf]

Supplementary material

Emerging Environmental Contaminants Targeting Cardiovascular Ion Channels: Exposure Effects, Underlying Mechanisms, and Implications for Cardiovascular Health Risks

Dingshan Zhan[1,†], Dan Li[1,†], Shulin Guo[1], Xuyang Chai[1], Rongkai Cao[1], Weicong Deng[1], Kaihan Wu[1], Yu Li[1], Suk Ying Tsang[2], Zongwei Cai[1,3,*], Zenghua Qi[1,*]

[1]Guangdong Key Laboratory of Environmental Catalysis and Health Risk Control, Guangdong-Hong Kong-Macao Joint Laboratory for Contaminants Exposure and Health, School of Environmental Science and Engineering, Guangdong University of Technology, Guangzhou 510006, China

[2]School of Life Sciences, The Chinese University of Hong Kong, Hong Kong, China

[3]Eastern Institute of Technology, Ningbo, 315100, China

[†]These authors contributed equally to this work.

*Corresponding author:

Zenghua Qi, Ph.D.

School of Environmental Science and Engineering

Guangdong University of Technology

Guangzhou, China

Tel: 020-39322292

Fax: 020-39322292

E-mail: zenghuaqi@gdut.edu.cn

Zongwei Cai, Ph.D.

College of Science

Eastern Institute of Technology,

Ningbo, China

E-mail: zwcai@eitech.edu.cn

Contents:

Supplementary Texts:

Text S1. Major ion channels of the cardiovascular system

Tables:

Table S1. Ion Channels in Cardiac Cells: Types, Functions, and Associated Cell Types

Figure:

Figure. S1. A flow diagram for the narrative literature review.

Text S1. Major ion channels of the cardiovascular system.

Cardiac ion channels play an essential role in maintaining the electrical and contractile functions of the heart, with precise regulation crucial for normal cardiac physiology [1]. Among these, sodium channels and exchangers, including VGSCs and NCX1, are key to the initiation and propagation of action potentials, ensuring cardiac excitability and rapid depolarization [2,3]. VGSCs, which are widely distributed in cardiac muscle, skeletal muscle, and neuronal axons, are composed of a large pore-forming α-subunit and one or more modulatory β-subunits. The α-subunit determines channel ion selectivity and voltage sensitivity, while the β-subunits modulate kinetic properties and channel expression [4]. Of the nine known α-subunit isoforms (Nav1.1–Nav1.9), Nav1.5—which is encoded by the *SCN5A* gene on chromosome 3p21—is the predominant form in cardiomyocytes and is critical for triggering the excitation-contraction coupling response [5]; mutations in *SCN5A* have been associated with a variety of arrhythmogenic disorders, such as long QT syndrome, Brugada syndrome, sick sinus syndrome, dilated cardiomyopathy, and atrial fibrillation [6-8]. NCX1, a bidirectional transporter consisting of nine transmembrane domains, regulates intracellular sodium and calcium levels by exchanging three Na^+ ions for one Ca^{2+} ion; with NCX1 being the principal isoform of the sodium–calcium exchanger in the heart, as it is indispensable for calcium homeostasis during the cardiac cycle. For this reason, the dysregulation of NCX1 has been implicated in arrhythmia, heart failure, myocardial ischemia/reperfusion injury, and diastolic dysfunction [9,10].

Calcium channels are equally important, as they mediate the influx of Ca^{2+} that is essential not only for the generation of action potentials but also for intracellular signaling and muscle contraction. These channels, which include pore-forming proteins permeable to Ca^{2+}, are classified into several types based on voltage sensitivity and activation kinetics; the L- and T-type channels

are the most prominent in cardiomyocytes [11]. L-type calcium channels, which are characterized by long open times and high-voltage activation, comprise a pore-forming α-subunit along with accessory β, δ, and γ subunits. These channels function as primary mediators of excitation-contraction coupling and contribute to early cardiac organogenesis, structural development, and sustained contractile function in both neonatal and adult hearts [12]. In contrast, T-type calcium channels—which open transiently—are primarily active in embryonic cardiomyocytes and pacemaker cells, where they facilitate atrioventricular conduction and contribute to the generation of pacemaker potentials. It is noteworthy that the expression of T-type calcium channels diminishes in the adult ventricular myocardium. Any dysregulation of calcium transport, whether at the level of the plasma membrane or within intracellular stores, can lead to calcium overload in mitochondria and nuclei, resulting in cellular dysfunction, heart failure, systolic dysfunction, arrhythmias, and even hypertension [13,14]. Several calcium release channels, such as RyRs and inositol trisphosphate receptors (IP_3Rs), complement these voltage-gated channels to regulate the release of Ca^{2+} from the sarcoplasmic reticulum [15]. There is strong evidence that RyR2 dysfunction is linked to heart failure and catecholaminergic polymorphic ventricular tachycardia, while IP_3R2 overexpression may cause cytoplasmic calcium overload and excessive cardiac contraction, ultimately leading to cardiomyocyte damage [16-18].

Potassium channels represent the most diverse class of cardiac ion channels and are critically responsible for repolarizing the membrane following depolarization; this function ensures the stability of the cardiac action potential. These channels can be categorized into several groups, including voltage-gated K^+ channels (Kv), inwardly rectifying K^+ channels (Kir), calcium-activated K^+ channels (K_{Ca}), and two-pore channels (K_{2P}) [19]. Kv channels, such as Kv4.3, contribute to the transient outward potassium current that aids in early repolarization, while Kv7.1

channels—which often function in complex with the KCNE1 subunit—mediate the delayed rectifier current that is essential for late repolarization [20,21]. Dysfunction in these channels is frequently associated with arrhythmogenic conditions like long QT syndrome [22]. Kir channels, notably Kir2.1, are integral for maintaining the resting membrane potential and stabilizing the action potential plateau, with abnormalities in these channels linked to cardiac arrhythmias [23]. Kir6.2, a core subunit of ATP-sensitive potassium channels, connects cellular metabolic status to electrical activity in cardiomyocytes, with dysfunction implicated in both diabetes mellitus and cardiovascular diseases [24]. K_{Ca} channels, e.g., $K_{Ca}1.1$, regulate vascular tone and muscle contraction by responding to intracellular calcium levels, with impairments in function potentially resulting in hypertension [25]. The overlapping functions and redundancy among these potassium channels contribute to the robustness of cardiac repolarization; nevertheless, any alterations or "remodeling" of these currents can predispose individuals to pathological states including cardiac hypertrophy, heart failure, atrial fibrillation, and ischemic heart disease [26-28].

Chloride channels, including voltage-gated chloride channels (ClCs) and the cystic fibrosis transmembrane conductance regulator, play a critical role in stabilizing the membrane potential, facilitating transepithelial transport, and maintaining intracellular pH and cell volume. ClC-2 and ClC-3 are the most heavily studied in cardiac tissues; ClC-2, which is predominantly expressed in atrial and ventricular myocytes, modulates action potential duration and intracellular pH, functions that are crucial for regulating cell volume and ionic balance. Dysfunctions in these chloride ion channels have been implicated in arrhythmogenesis and myocardial hypertrophy [29,30].

Non-selective cation channels, particularly those of the TRP family, further expand the repertoire of ionic conductance in the heart. TRP channels, which are divided into several subfamilies such as TRPC ("C" for canonical), TRPV ("V" for vanilloid), TRPM ("M" for

melastatin), TRPN ("N" for no mechanoreceptor potential C), and TRPA ("A" for ankyrin), are involved in various cellular processes including contraction, proliferation, and adaptive remodeling [31-33]. In the cardiac context, TRPC3, TRPC6, and TRPM4 are known to influence action potential duration, conduction velocity, and pacemaker activity [34-36]. Additionally, hyperpolarization-activated cyclic nucleotide-gated (HCN) channels, which generate the pacemaker current (I_f), are critical in the sinoatrial node; more specifically, mutations in *HCN4* have been linked to inappropriate sinus tachycardia and an increased risk of adult heart disease [37]. Mechanical-electrical coupling, which is mediated by stretch-activated channels, further exemplifies the complexity of ion channel regulation, as mechanical forces such as atrial stretch can induce changes in excitability and contribute to arrhythmias like atrial fibrillation by promoting sustained calcium leakage from the sarcoplasmic reticulum [38,39].

Table S1. Ion Channels in Cardiac Cells: Types, Functions, and Associated Cell Types.

Ion Type	Type of Ion Channel	Subtype	Function	Cardiac Cell Types
Sodium (Na^+)	Voltage-gated sodium channels	$Na_V1.5$	Rapid depolarization (Phase 0 of cardiac action potential)	Cardiomyocytes (atrial, ventricular, Purkinje fibers)
	Sodium-calcium exchanger	NCX1	Exchange of 3 Na^+ for 1 Ca^{2+}; Regulation of Ca^{2+} homeostasis	Cardiomyocytes (atrial, ventricular)
Calcium (Ca^{2+})	Voltage-gated calcium channels	L-type ($Ca_V1.2$)	Plateau phase (Phase 2) and excitation-contraction coupling	Cardiomyocytes (atrial, ventricular), SA node (pacemaker cells), AV node
		T-type	Pacemaker activity in	SA node (pacemaker

		($Ca_V3.1/3.2$)	SA and AV nodes	cells), AV node, atrial cardiomyocytes
	Calcium release channels	RyR	Release of Ca^{2+} from SR during excitation-contraction coupling	Cardiomyocytes (atrial, ventricular)
		IP_3 receptors	Release of Ca^{2+} from SR in response to IP_3 signaling	Cardiomyocytes (less prominent than RyR)
Potassium (K^+)	Voltage-gated potassium channels	Kv4.3 (I_{to})	Transient outward current (Phase 1 repolarization)	Cardiomyocytes (atrial, ventricular)
		Kv7.1 (I_{Ks})	Slow delayed rectifier K^+ current (Phase 3 repolarization)	Cardiomyocytes (atrial, ventricular), SA node (pacemaker cells), AV node
		Kv11.1 (I_{Kr})	Rapid delayed rectifier K^+ current (Phase 3 repolarization)	Cardiomyocytes (atrial, ventricular), SA node (pacemaker cells), AV node
	Inwardly rectifying potassium channels	Kir2.1 (I_{K1})	Maintenance of resting membrane potential (Phase 4)	Cardiomyocytes (atrial, ventricular)
		Kir6.2 (K_{ATP})	ATP-sensitive K^+ current; Protection of tissue during ischemia	Cardiomyocytes (atrial, ventricular), vascular smooth muscle
	Calcium-activated potassium channels	BK ($K_{Ca}1.1$)	Regulation of vascular tone and smooth muscle relaxation	Vascular smooth muscle cells

Chloride (Cl^-)	Chloride channels	ClC-2	Stabilization of resting membrane potential	Cardiomyocytes (atrial, ventricular)
		ClC-3	Cell volume regulation	Cardiomyocytes (atrial, ventricular)
Non-Selective Cations	Hyperpolarization-activated cyclic nucleotide-gated channels	HCN4	Pacemaker current (I_f) in SA and AV nodes	SA node (pacemaker cells), AV node, Purkinje fibers
	Transient receptor potential channels	TRPC	Ca^{2+} influx in response to stress or signaling	Cardiomyocytes (atrial, ventricular), vascular smooth muscle
		TRPV	Mechanical and osmotic sensing	Cardiomyocytes (atrial, ventricular), vascular smooth muscle
	Stretch-activated channels	N/A	Response to mechanical stretch; regulation of Na^+ and Ca^{2+} influxes	Cardiomyocytes (atrial, ventricular), vascular smooth muscle

Abbreviations: AV, atrioventricular; BK, big conductance calcium-activated potassium channel; I_{K1}, inward rectifier potassium current; I_{Kr}, rapid delayed rectifier potassium current; I_{Ks}, slow delayed rectifier potassium current; IP_3, inositol 1,4,5-trisphosphate; K_{ATP}, ATP-sensitive potassium channel; SA, sinoatrial; SR, sarcoplasmic reticulum.

Figure S1. A flow diagram for the narrative literature review.

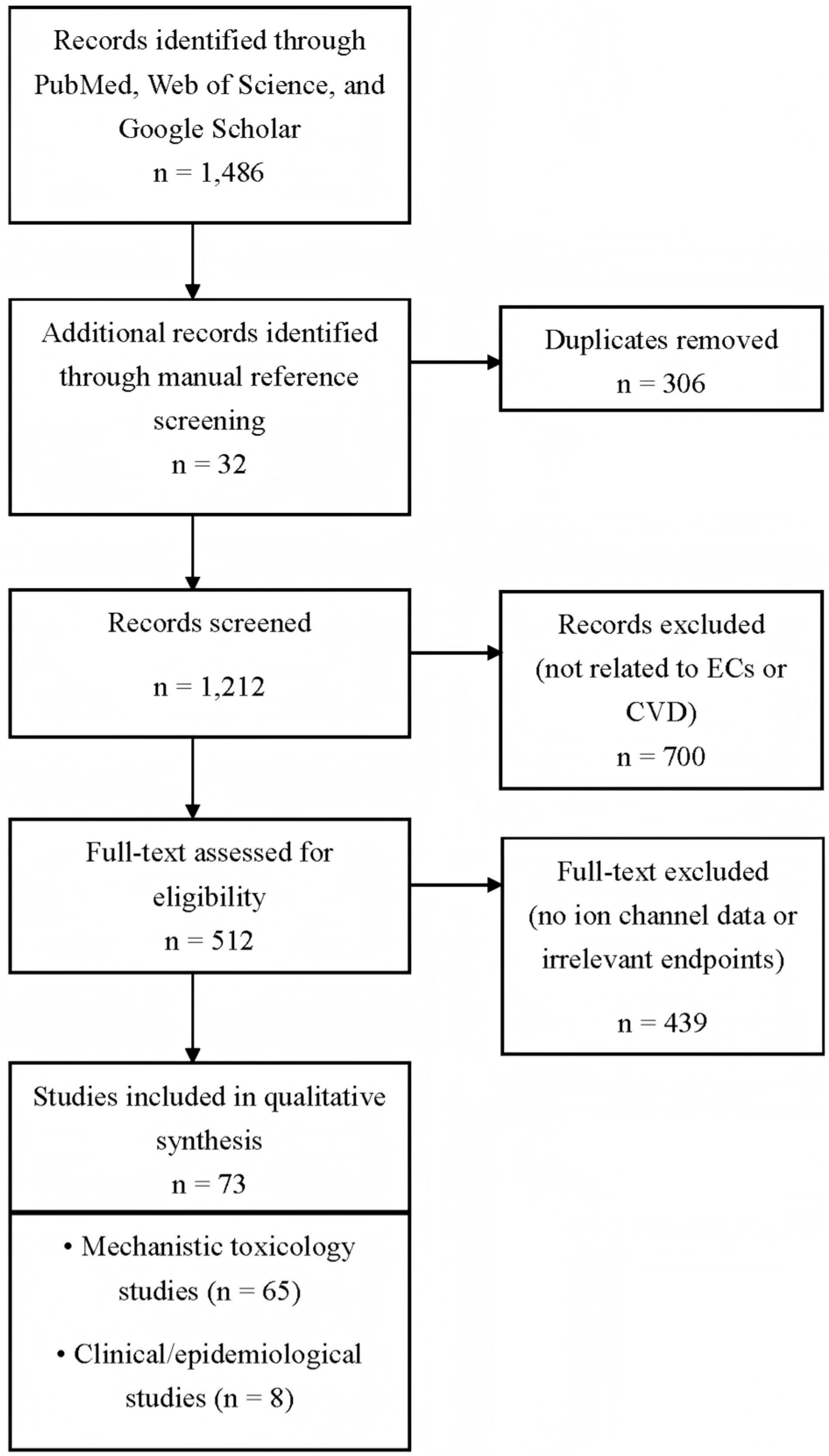

References:

1. Alsaloum, M.; Dib-Hajj, S.D.; Page, D.A.; Ruben, P.C.; Krainer, A.R.; Waxman, S.G. Voltage-gated sodium channels in excitable cells as drug targets. *Nature Reviews Drug Discovery* **2025**, *24*, 358-378, doi:10.1038/s41573-024-01108-x.
2. Priest; McDermott. Cardiac ion channels. *Channels* **2015**, *9*, 352-359, doi:10.1080/19336950.2015.1076597.
3. Salvage, S.C.; Jeevaratnam, K.; Huang, C.L.H.; Jackson, A.P. Cardiac sodium channel complexes and arrhythmia: structural and functional roles of the β1 and β3 subunits. *Journal of Physiology-London* **2023**, *601*, 923-940, doi:10.1113/jp283085.
4. Jiang, D.; Shi, H.; Tonggu, L.; El-Din, T.M.G.; Lenaeus, M.J.; Zhao, Y.; Yoshioka, C.; Zheng, N.; Catterall, W.A. Structure of the Cardiac Sodium Channel. *Cell* **2020**, *180*, 122-+, doi:10.1016/j.cell.2019.11.041.
5. Han, D.; Tan, H.; Sun, C.; Li, G. Dysfunctional Nav1.5 channels due to SCN5A mutations. *Experimental Biology and Medicine* **2018**, *243*, 852-863, doi:10.1177/1535370218777972.
6. Pierre, M.; Djemai, M.; Pouliot, V.; Poulin, H.; Gollob, M.H.; Chahine, M. Exploring SCN5A variants associated with atrial fibrillation in atrial cardiomyocytes derived from human induced pluripotent stem cells: A characterization study. *Heart rhythm* **2024**, doi:10.1016/j.hrthm.2024.09.013.
7. Qi, M.; Ma, S.; Liu, J.; Liu, X.; Wei, J.; Lu, W.-J.; Zhang, S.; Chang, Y.; Zhang, Y.; Zhong, K.; et al. In Vivo Base Editing of Scn5a Rescues Type 3 Long QT Syndrome in Mice. *Circulation* **2024**, *149*, 317-329, doi:10.1161/circulationaha.123.065624.
8. Zhang; Barajas-Martinez; Xia; Li; Capra; Clatot; Chen, G.-X.; Chen, X.; Yang, B.; Jiang, H.; et al. Distinct Features of Probands With Early Repolarization and Brugada Syndromes Carrying SCN5A Pathogenic Variants. *Journal of the American College of Cardiology* **2021**, *78*, 1603-1617, doi:10.1016/j.jacc.2021.08.024.
9. Wang; Song; Yuan; Li; Abu-Taha, I.; Heijman, J.; Sun, L.; Dobrev, S.; Kamler, M.; Xie, L.; et al. Downregulation of FKBP5 Promotes Atrial Arrhythmogenesis. *Circulation Research* **2023**, *133*, E1-E16, doi:10.1161/circresaha.122.322213.
10. Xue, J.; Zeng, W.; Han, Y.; John, S.; Ottolia, M.; Jiang, Y. Structural mechanisms of the human cardiac sodium-calcium exchanger NCX1. *Nature Communications* **2023**, *14*, doi:10.1038/s41467-023-41885-4.
11. Shah, K.; Seeley, S.; Schulz, C.; Fisher, J.; Rao, S.G. Calcium Channels in the Heart: Disease States and Drugs. *Cells* **2022**, *11*, doi:10.3390/cells11060943.
12. Xu; Sun; Xie; Mou, S.; Zhang, D.; Zhu, J.; Xu, P. Advances in L-Type Calcium Channel Structures, Functions and Molecular Modeling. *Current Medicinal Chemistry* **2021**, *28*, 514-524, doi:10.2174/0929867327666200714154059.
13. Morciano, G.; Rimessi, A.; Patergnani, S.; Vitto, V.A.M.; Danese, A.; Kahsay, A.; Palumbo, L.; Bonora, M.; Wieckowski, M.R.; Giorgi, C.; et al. Calcium dysregulation in heart diseases: Targeting calcium channels to achieve a correct calcium homeostasis. *Pharmacological Research* **2022**, *177*, doi:10.1016/j.phrs.2022.106119.
14. Walkon, L.L.; Strubbe-Rivera, J.O.; Bazil, J.N. Calcium Overload and Mitochondrial

Metabolism. *Biomolecules* **2022**, *12*, doi:10.3390/biom12121891.
15. Woll, K.A.; Van Petegem, F. Calcium-release channels: structure and function of IP3 receptors and ryanodine receptors. *Physiological Reviews* **2022**, *102*, 209-268, doi:10.1152/physrev.00033.2020.
16. Dridi, H.; Santulli, G.; Gambardella, J.; Jankauskas, S.S.; Yuan, Q.; Yang, J.; Reiken, S.; Wang, X.; Wronska, A.; Liu, X.; et al. IP3 receptor orchestrates maladaptive vascular responses in heart failure. *The Journal of Clinical Investigation* **2022**, *132*, doi:10.1172/JCI152859.
17. Kushnir, A.; Wajsberg, B.; Marks, A.R. Ryanodine receptor dysfunction in human disorders. *Biochimica Et Biophysica Acta-Molecular Cell Research* **2018**, *1865*, 1687-1697, doi:10.1016/j.bbamcr.2018.07.011.
18. Piamsiri, C.; Fefelova, N.; Pamarthi, S.H.; Gwathmey, J.K.; Chattipakorn, S.C.; Chattipakorn, N.; Xie, L.-H. Potential Roles of IP3 Receptors and Calcium in Programmed Cell Death and Implications in Cardiovascular Diseases. *Biomolecules* **2024**, *14*, doi:10.3390/biom14101334.
19. Grandi, E.; Sanguinetti, M.C.; Bartos, D.C.; Bers, D.M.; Ye, C.-I.; Chiamvimonvat, N.; Colecraft, H.M.; Delisle, B.P.; Heijman, J.; Navedo, M.F.; et al. Potassium channels in the heart: structure, function and regulation. *Journal of Physiology-London* **2017**, *595*, 2209-2228, doi:10.1113/jp272864.
20. Ma, D.; Zhao, C.; Wang, X.; Li, X.; Zha, Y.; Zhang, Y.; Fu, G.; Liang, P.; Guo, J.; Lai, D. Structural basis for the gating modulation of Kv4.3 by auxiliary subunits. *Cell Research* **2022**, *32*, 411-414, doi:10.1038/s41422-021-00608-4.
21. Sanguinetti, M.C.; Seebohm, G. Physiological Functions, Biophysical Properties, and Regulation of KCNQ1 (KV7.1) Potassium Channels. *Advances in experimental medicine and biology* **2021**, *1349*, 335-353, doi:10.1007/978-981-16-4254-8_15.
22. Egly, C.; Barny, L.; Plate, L.; Knollmann, B. Title: Protein interaction profiling of the Kv11.1 potassium channel reveals new therapeutic targets for Long QT Syndrome. *Circulation* **2024**, *150*, doi:10.1161/circ.150.suppl_1.4136861.
23. Reilly, L.; Eckhardt, L.L. Cardiac potassium inward rectifier Kir2: Review of structure, regulation, pharmacology, and arrhythmogenesis. *Heart Rhythm* **2021**, *18*, 1423-1434, doi:10.1016/j.hrthm.2021.04.008.
24. Wang, Z.; Bian, W.; Yan, Y.; Zhang, D.-M. Functional Regulation of KATP Channels and Mutant Insight Into Clinical Therapeutic Strategies in Cardiovascular Diseases. *Frontiers in Pharmacology* **2022**, *Volume 13 - 2022*, doi:10.3389/fphar.2022.868401.
25. Pineda, S.; Nikolova-Krstevski, V.; Leimena, C.; Atkinson, A.J.; Altekoester, A.-K.; Cox, C.D.; Jacoby, A.; Huttner, I.G.; Ju, Y.-K.; Soka, M.; et al. Conserved Role of the Large Conductance Calcium-Activated Potassium Channel, KCa1.1, in Sinus Node Function and Arrhythmia Risk. *Circulation-Genomic and Precision Medicine* **2021**, *14*, 230-242, doi:10.1161/circgen.120.003144.
26. Burg, S.; Attali, B. Targeting of Potassium Channels in Cardiac Arrhythmias. *Trends in Pharmacological Sciences* **2021**, *42*, 491-506, doi:10.1016/j.tips.2021.03.005.
27. Bossuyt, J.; Borst, J.M.; Verberckmoes, M.; Bailey, L.R.J.; Bers, D.M.; Hegyi, B. Protein Kinase D1 Regulates Cardiac Hypertrophy, Potassium Channel Remodeling, and

Arrhythmias in Heart Failure. *Journal of the American Heart Association* **2022**, *11*, e027573, doi:10.1161/JAHA.122.027573.
28. Li, J.; Shen, R.; Reddy, B.; Perozo, E.; Roux, B. Mechanism of C-type inactivation in the hERG potassium channel. *Science Advances* **2021**, *7*, eabd6203, doi:10.1126/sciadv.abd6203.
29. Harraz, O.F.; Delpire, E. Recent insights into channelopathies. *Physiological Reviews* **2024**, *104*, 23-31, doi:10.1152/physrev.00022.2023.
30. Voronina, Y.A.; Karhov, A.M.; Kuzmin, V.S. Structural Basis and Molecular Mechanisms of Cl– Transmembrane Transport in Cardiomyocytes. *Moscow University Biological Sciences Bulletin* **2024**, *79*, S17-S32, doi:10.3103/s0096392524600741.
31. Gwanyanya, A.; Mubagwa, K. Emerging role of transient receptor potential (TRP) ion channels in cardiac fibroblast pathophysiology. *Frontiers in Physiology* **2022**, *13*, doi:10.3389/fphys.2022.968393.
32. Lezama-García, K.; Mota-Rojas, D.; Pereira, A.M.F.; Martínez-Burnes, J.; Ghezzi, M.; Domínguez, A.; Gómez, J.; de Mira Geraldo, A.; Lendez, P.; Hernández-Ávalos, I.; et al. Transient Receptor Potential (TRP) and Thermoregulation in Animals: Structural Biology and Neurophysiological Aspects. *Animals* **2022**, *12*, doi:10.3390/ani12010106.
33. Zhang, M.; Ma, Y.; Ye, X.; Zhang, N.; Pan, L.; Wang, B. TRP (transient receptor potential) ion channel family: structures, biological functions and therapeutic interventions for diseases. *Signal Transduction and Targeted Therapy* **2023**, *8*, doi:10.1038/s41392-023-01464-x.
34. Chubanov, V.; Koettgen, M.; Touyz, R.M.; Gudermann, T. TRPM channels in health and disease. *Nature Reviews Nephrology* **2024**, *20*, 175-187, doi:10.1038/s41581-023-00777-y.
35. Matsumoto, T.; Taguchi, K.; Kobayashi, T. Role of TRPV4 on vascular tone regulation in pathophysiological states. *European Journal of Pharmacology* **2023**, *959*, doi:10.1016/j.ejphar.2023.176104.
36. Tang, N.; Tian, W.; Ma, G.-Y.; Xiao, X.; Zhou, L.; Li, Z.-Z.; Liu, X.-X.; Li, C.-Y.; Wu, K.-H.; Liu, W.; et al. TRPC channels blockade abolishes endotoxemic cardiac dysfunction by hampering intracellular inflammation and Ca2+ leakage. *Nature Communications* **2022**, *13*, doi:10.1038/s41467-022-35242-0.
37. Cámara-Checa, A.; Perin, F.; Rubio-Alarcón, M.; Dago, M.; Crespo-García, T.; Rapún, J.; Marín, M.; Cebrián, J.; Gómez, R.; Bermúdez-Jiménez, F.; et al. A gain-of-function HCN4 mutant in the HCN domain is responsible for inappropriate sinus tachycardia in a Spanish family. *Proceedings of the National Academy of Sciences* **2023**, *120*, e2305135120, doi:10.1073/pnas.2305135120.
38. Medvedev, R.Y.; Afolabi, S.O.; Turner, D.G.P.; Glukhov, A.V. Mechanisms of stretch-induced electro-anatomical remodeling and atrial arrhythmogenesis. *Journal of Molecular and Cellular Cardiology* **2024**, *193*, 11-24, doi:10.1016/j.yjmcc.2024.05.011.
39. Zhang; Qi; Li; He, W.-J.; Gao, X.-H.; Zhang, Y.; Sun, X.; Tong, J.; Zhang, J.; Deng, X.-L.; et al. Stretch-induced sarcoplasmic reticulum calcium leak is causatively associated with atrial fibrillation in pressure-overloaded hearts. *Cardiovascular Research* **2021**, *117*, 1091-1102, doi:10.1093/cvr/cvaa163.
